# A Comparative Study of Sample Preparation for Staining and Immunodetection of Plant Cell Walls by Light Microscopy

**DOI:** 10.3389/fpls.2017.01505

**Published:** 2017-08-29

**Authors:** Yves Verhertbruggen, Jesse L. Walker, Fabienne Guillon, Henrik V. Scheller

**Affiliations:** ^1^Joint BioEnergy Institute, Lawrence Berkeley National Laboratory Emeryville, CA, United States; ^2^Environmental Genomics and Systems Biology Division, Lawrence Berkeley National Laboratory Berkeley, CA, United States; ^3^Institut National de la Recherche Agronomique, UR 1268 Nantes, France; ^4^Department of Plant and Microbial Biology, University of California, Berkeley Berkeley, CA, United States

**Keywords:** cell wall-degrading enzyme, embedding, fluorescence, immunodetection, microscopy, polysaccharide, sectioning, toluidine blue

## Abstract

Staining and immunodetection by light microscopy are methods widely used to investigate plant cell walls. The two techniques have been crucial to study the cell wall architecture *in planta*, its deconstruction by chemicals or cell wall-degrading enzymes. They have been instrumental in detecting the presence of cell types, in deciphering plant cell wall evolution and in characterizing plant mutants and transformants. The success of immunolabeling relies on how plant materials are embedded and sectioned. Agarose coating, wax and resin embedding are, respectively, associated with vibratome, microtome and ultramicrotome sectioning. Here, we have systematically carried out a comparative analysis of these three methods of sample preparation when they are applied for cell wall staining and cell wall immunomicroscopy. In order to help the plant community in understanding and selecting adequate methods of embedding and sectioning for cell wall immunodetection, we review in this article the advantages and limitations of these three methods. Moreover, we offer detailed protocols of embedding for studying plant materials through microscopy.

## Introduction

Plant cell walls are fundamental to plant biology and have a strong impact on the use of plant products in industrial processes. Studying cell walls helps to adapt crops to climate change, to improve the production of biofuels, and to increase the productivity of food and feed, which is necessary to support the incoming growth of the global population. The efficiency of plant cell wall staining and immunomicroscopy has made both techniques widely used for plant cell wall analysis. For example, immunolabeling procedures have been crucial to study the cell wall architecture *in planta* (e.g., Knox et al., 1990; Majewska-Sawka et al., 2004; Guillemin et al., 2005; Xue et al., 2013) and its deconstruction by chemicals or cell wall-degrading enzymes (Marcus et al., 2008; Hervé et al., 2010). Both staining and immunodetection of plant polysaccharides enable to focus on specific cell types (Hall et al., 2013; Eeckhout et al., 2014) and immunolabelings even allow to isolate certain cell types from their neighbor cells (Verhertbruggen et al., 2009b). As a highly sensitive technique, immunolabeling is thus a powerful method to investigate plant histology. Cell wall immunolabeling has contributed in evaluating plant cell wall evolution and in redefining plant taxonomy (Leroux et al., 2011, 2015). It has been instrumental in detecting cell wall alterations in plant mutants and transformants (e.g., Oomen et al., 2002; Freshour et al., 2003; Harholt et al., 2012; Zabotina et al., 2012; Salazar-Iribe et al., 2016) and has been a powerful technique to gain knowledge about the biological and physiological functions of cell wall polymers (e.g., Willats et al., 1999; Leboeuf et al., 2005; Domozych et al., 2014). To date, more than 200 probes directed to plant cell wall components, mostly monoclonal antibodies, are commercially available from three sources: CarboSource, PlantProbes and Biosupplies. Many additional probes, including carbohydrate-binding modules, that are not currently commercially available have been generated by various researchers and may be available upon request. Most indirect immunolabelings are based on a “two step” procedure where the primary probe that targets a specific polysaccharidic feature is detected by the use of a secondary antibody coupled to a fluorochrome. Although protocols for immunolabeling have been extensively described (Willats et al., 2002; Herve et al., 2011; Avci et al., 2012; Lee and Knox, 2014), no direct comparison has been provided on the methods of embedding and their associated modes of sectioning in regards to plant cell wall staining and immunodetection *in planta*. Whereas the success of cell wall staining and immunolabeling depends on these methods and how they are designed, information regarding their advantages and disadvantages is lacking. Sample preparation determines the quality and the quantity of material obtained for histological analysis and immunomicroscopy, and defines how realistic an experiment can be done in respect with an allocated time frame. To maximize the chance of success in immunomicroscopy, it is therefore important to know which method of sample preparation is most appropriate for the applications or the goals one desires to achieve. Three methods of sample preparation are commonly used. As it can be applied to both light and electron microscopy, the sample preparation by resin-embedding and ultramicrotome sectioning is a widely used method (Yin et al., 2011; Domozych et al., 2014; Moro et al., 2016). The Paul Knox laboratory, who has generated the collection of JIM and LM antibodies, preferentially opts for wax embedding with microtome-sectioning and recommends this technique (Willats et al., 2002; Herve et al., 2011). In the meantime, the coating in agarose of non-embedded samples followed by sectioning with a vibratome has recently been shown to be effective for studying plant cell walls of *Arabidopsis thaliana* (Hall et al., 2013; Verhertbruggen et al., 2013), a genetic model extensively used to study plant cell walls and their biosynthesis (see, for instance, the review of Mohnen, 2008). This last method appears to be particularly convenient for comparative analysis of stem samples (Hall et al., 2013; Verhertbruggen et al., 2013).

Here, while using equivalent stem samples of *Arabidopsis thaliana* as plant material, we have systematically compared these three methods of coating or embedding (agarose, wax, and resin) and their respective method of sectioning (vibratome, microtome, and ultramicrotome). We present the advantages and the limitations of these techniques when applied for cell wall staining and immunomicroscopy. We demonstrate how they impact on primary fluorescence (also known as autofluorescence) detection and how they can affect the accessibility of antibodies and cell wall-degrading enzymes to targeted polymers. We discuss which procedure is the most adapted for staining, primary fluorescence study, immunolabeling and analysis of chemical and enzymatic treatments. Moreover, we also offer detailed protocols of embedding for studying plant materials through microscopy.

## Materials and methods

### Preparation of plant material for immunomicroscopy

Here, our focus is the preparation of plant material for plant cell wall staining and immunolabeling. Methods of plant sample preparation have been produced for other type of microscope analysis and are discussed elsewhere (see for examples Hayat, 1981; Soukup and Tylova, 2014; Yeung and Chan, 2015). For immunofluorescence microscopy of plant cell walls, the first step consists in incubating the plant material in a fixative solution that commonly contains 4% (w/v) paraformaldehyde or a mixture of paraformaldehyde with glutaraldehyde (Pattathil et al., 2010; Ralet et al., 2010; Yin et al., 2011; Domozych et al., 2014). Compared to paraformaldehyde, glutaraldehyde better preserves the cellular structure (Hayat, 1981). However, glutaraldehyde does not penetrate the tissues as rapidly as paraformaldehyde and, most importantly, it induces loss of antigenicity (Hayat, 1981; Marttila and Santén, 2007). Since both aldehydes leads to the reticulation of proteins, carbohydrates are unlikely to be directly modified by these fixatives. On the other hand, since the reticulation of proteins induces steric hindrances, it is possible that crosslinking of proteins may indirectly affect the accessibility of some carbohydrate epitopes. Cell wall immunomicroscopy assays can be carried out on samples fixed with up to 2.5% (w/v) glutaraldehyde (Freshour et al., 2003). Hawes and Satiat-Jeunemaitre (2001) have determined in their study that its use should ideally not exceed 1% of the fixative solution. To preserve the integrity of the samples and thus their antigenicity, it is important to use a fixative solution that has a minimal effect on the osmosis of the tissues. For our samples, we usually used a PEM (50 mM piperazine-N,N'-bis[2-ethanesulphonic acid], 5 mM EGTA, 5 mM MgSO_4_; pH 6.9) buffer as it also provides the advantages of stabilizing microtubules (Arakawa and Timasheff, 1984; Wick, 1993). Alternative methods of fixation are presented in Marttila and Santén (2007). The penetration of the fixative chemical depends on the plant material and can occur within 1 h (Vitha et al., 2000). For practical reasons, we usually section pieces of ca. 0.5 cm^3^ (0.5 cm being the maximal length for each direction) and incubate them overnight in 4% (v/v) paraformaldehyde in PEM buffer at 4°C. Depending of the plant material and the aim of the study, the samples can be stored in this condition for longer period. In the present study, we have applied the same procedure of fixation to the pieces of *A. thaliana* stem (4% (v/v) paraformaldehyde in PEM buffer at 4°C for about 16 h) used for the three different methods of sample preparation. The fixation was terminated by washing the samples thrice with the buffer used to fix the samples—here the PEM buffer—(3 × 5 min) and once with deionized water for 5 min. The samples were then immediately coated or embedded as detailed in the following paragraphs.

### Agarose-embedding and vibratome sectioning

Styrofoam, carrot roots, cork or agarose are commonly used as support to mount and section plant materials with vibratome (Ruzin, 1999). Soft samples, such as *A. thaliana* inflorescence or rice cotyledon, are often easily sectioned when embedded in agarose. For such plant materials, we pour freshly made 7% (w/v) agarose into conical centrifuge plastic tubes and let the solution cool down for 1 min. As soon as the solution starts to form a gel, the plant material is inserted into the agarose and the bottom of the tube is cut off once the agarose has completely solidified. The cylinder of agarose is extracted and glued on a specimen disk using an instant tissue adhesive based on cyanoacrylate (Loctite, SuperGlue 3 Power Flex, Belgium). Prior to filling the buffer tray with distilled water, the specimen disks are fixed on the buffer tray and abundantly rinsed with tap water to remove the excess of glue. The section quality will depend on the plant material, the blade used for sectioning and the settings of the vibratome (Ruzin, 1999). In general, we obtain a good proportion of excellent 60 μm thick sections using disposable razor blade (double edge, polytetrafluorethylene (PTFE) coated stainless steel) angled at ca. 5° and moving at 2.5 mm/s with an oscillation/sectioning frequency of 80 Hz. The sections are released from the agarose, collected and can be stored in distilled water for a few days at 4°C. The vibratomes used here were a Leica VT1000s and a Microm Microtech HM 650 V.

### Wax-embedding and microtome sectioning

The protocol presented here is an advanced method based on former protocols using Steedman's wax embedding for immunomicroscopy (Vitha et al., 2000; Herve et al., 2011). It is relatively short and we recommend using it as a starting point. If the results are not satisfying, we advise to first extend the incubation times during the steps of dehydration and infiltration. Compared to other protocols, our protocol relies on the use of bees wax. Its advantages are that, unlike regular Steedman's wax, an ethanol-soluble low melting point polyester wax (37°C), that is often brittle and hard to handle in a warm environment, bees wax is softer and with a melting point above 100°C. The blocks of Steedman's wax that contains 7% bees wax are easy to manipulate, provide a high ratio of excellent sections, and are easy to store.

Both Steedman's and bees wax are commercially available (EMS, USA and Sigma Aldrich, USA, respectively). However, Steedman's wax can easily be prepared from polyethylene glycol 400 distearate and 1-hexadecanol (Sigma Aldrich, USA). The chemicals are slowly melted at a ratio of 9:1 at 65°C with continual stirring. Once melted, the solution is vigorously shaken and poured in a container wrapped with aluminum fold to solidify. The wax solution can be stored at room temperature (RT) and melted at 37°C when required.

After fixation in formaldehyde and the series of washes, plant materials are dehydrated in a graded series of aqueous ethanol solution and infiltrated with Steedman's wax as described in Table 1. Once infiltrated in wax, the samples are disposed in base molds (VWR international, USA) which are filled with a solution of Steedman's wax containing 7% bees wax and covered with an embedding cassette. Here, particular attention must be taken to orientate the plant material to optimize its sectioning. When the molds are solidified, the blocks are ready to be sectioned to a thickness of 12 μm with a microtome, for example Microm Microtech HM325, using a “low profile” disposable microtome blade coated with PTFE (EMS, USA). The blade is replaced by a new one as soon as the section quality starts to decline. For frequent users, it is recommended to consider obtaining a reusable knife. Once cut, the sections are collected onto glass slides coated with polylysine (VWR international, USA), and a drop of distilled water is applied at the surface of the slides that are held slightly inclined. This will allow the sections to spread and will promote their adhesion onto the slides. Water must be pure as contaminated distilled water can result in a loss of adherence of the sections onto the slides. After air-drying overnight at RT, the sections are dewaxed and rehydrated in a graded series of aqueous solution as indicated in Table 2. The sections are dried at RT and can then be immediately used for analysis or stored in dry conditions for years.

**Table 1:** Procedure for dehydrating and infiltrating plant material with Steedman's wax.

**Step**	**Solution**	**Incubation time**	**Temperature**
a	30% (v/v) ethanol/dH_2_O	30 min	4°C
b	50% (v/v) ethanol/dH_2_O	30 min	4°C
c	70% (v/v) ethanol/dH_2_O	30 min	4°C
d	90% (v/v) ethanol/dH_2_O	30 min	4°C
e	100% (v/v) ethanol/dH_2_O	30 min	4°C
f	1:1 (v/v) wax/ethanol	Overnight	37°C
g	100% Steedman's wax	60 min	37°C
h	100% Steedman's wax	60 min	37°C

**Table 2:** Procedure for dewaxing and rehydratating sections embedded in Steedman's wax.

**Step**	**Solution**	**Incubation time**	**Temperature**
a	100% ethanol	10 min	RT
b	100% ethanol	10 min	RT
c	100% ethanol	10 min	RT
d	90% (v/v) ethanol	10 min	RT
e	50% (v/v) ethanol	10 min	RT
g	100% dH_2_O	10 min	RT
h	100% dH_2_O	90 min	RT

### London resin white-embedding and ultramicrotome sectioning

Many protocols of embedding with London Resin (LR) White are available for plant material (see for examples: Hayat, 1981; Marttila and Santén, 2007; Lee and Knox, 2014). We have optimized two protocols for embedding soft and hard plant materials using the hydrophilic acrylic LR White hard grade (EMS, USA). Although it is advised by the producer to avoid the use of LR White resin below −15°C, we have obtained, similarly to Bush and McCann (1999), excellent sections by following the procedure described in Table 3 and termed “soft.” This protocol has already been briefly described in Yin et al. (2011) and provided excellent results for soft plant materials such as the apical region of *A. thaliana* stem or stem sections from *A. thaliana* mutant plants displaying severe dwarf phenotypes. The second protocol, termed “hard,” mostly carried out at room temperature, has provided excellent sections of silicate-rich rice stem nodes and internodes (Chen et al., 2013) as well as thick and strongly lignified inflorescence stem of *A. thaliana* (Verhertbruggen et al., 2009b; Yang et al., 2013). It is important to note that, when the aim is to study the samples by both light and electron microscopy, the fixation of the samples must be carried out with a mixture of paraformaldehyde and glutaraldehyde (Marttila and Santén, 2007).

**Table 3:** Steps for the dehydration and the embedding with London Resin White of plant materials prepared for the analysis of plant cell walls by immunomicroscopy.

	**Soft**			**Hard**		
**Step**	**Solution**	**Incubation time**	**Temp**.	**Solution**	**Incubation time**	**Temp**.
**WASHES AFTER FIXATION**						
a	Buffer solution used to fix the samples	3 × 5 min	4°C	Buffer solution used to fix the samples	3 × 5 min	RT
b	dH_2_O	5 min	4°C	dH_2_O	5 min	RT
**DEHYDRATATION**						
c	10% (v/v) ethanol/dH_2_O	10 min	4°C	10% (v/v) ethanol/dH_2_O	10 min	RT
d	20% (v/v) ethanol/dH_2_O	10 min	0°C	20% (v/v) ethanol/dH_2_O	10 min	RT
e	30% (v/v) ethanol/dH_2_O	10 min	−20°C	30% (v/v) ethanol/dH_2_O	10 min	RT
f	50% (v/v) ethanol/dH_2_O	10 min	−20°C	50% (v/v) ethanol/dH_2_O	10 min	RT
g	70% (v/v) ethanol/dH_2_O	30 min	−20°C	70% (v/v) ethanol/dH_2_O	30 min	RT
h	90% (v/v) ethanol/dH_2_O	30 min	−20°C	90% (v/v) ethanol/dH_2_O	30 min	RT
i	100% ethanol	30 min	−20°C	100% ethanol	30 min	RT
j	100% ethanol	30 min	−20°C	100% ethanol	O/N	RT
k	100% ethanol	30 min	−20°C			
**INFILTRATION**						
l	10% (v/v) LR White/ethanol	1 h	−20°C	10% (v/v) LR White/ethanol	1 h	RT
m	30% (v/v) LR White/ethanol	1 h	−20°C	30% (v/v) LR White/ethanol	1 h	RT
n	50% (v/v) LR White/ethanol	1 h	−20°C	50% (v/v) LR White/ethanol	1 h	RT
o	70% (v/v) LR White/ethanol	1 h	−20°C	70% (v/v) LR White/ethanol	1 h	RT
p	90% (v/v) LR White/ethanol	1 h	−20°C	90% (v/v) LR White/ethanol	1 h	RT
q	100% LR White	8 h	−20°C	100% LR White	O/N	4°C
r	100% LR White	8 h	−20°C	100% LR White	8 h	4°C
s	100% LR White	8 h	−20°C			

*On the left, the protocol termed “soft” is used for embedding soft plant materials such as the apical region of A. thaliana stems. On the right, the protocol termed “hard” is used for embedding hard plant materials such as strongly lignified A. thaliana stems or silicate-rich rice stem internodes. O/N, overnight; RT, room temperature, LR, London resin, dH_2_O, distilled water*.

After fixation, plant materials were dehydrated in a graded series of aqueous ethanol solution and infiltrated with London Resin White as shown in Table 3. After complete polymerization in capsules with absence of oxygen at 55/60°C, LR White embedded materials are sectioned with a diamond knife mounted on a Leica UC6 ultramicrotome to obtain semi-thin sections of either 0.5 or 1 μm of thickness. The sections are collected on 10 well-slides (EMS, USA) coated with Vectabond reagent (Vector Laboratories, UK). For each well, ca. 5 sections are loaded in a drop of ultra-pure water. The sections are air-dried overnight at RT. The slides can then be immediately used for analysis or stored in dry conditions for years.

### Indirect immunolabeling of plant cell walls

Detailed protocols of indirect immunolabeling of plant cell walls are already available (e.g., (Willats et al., 2002; Herve et al., 2011). Here, we will describe preliminary steps that can be required for the preparation of samples in suspension or adhered on a slide. Moreover, we advise extra steps for those who desire to reduce the autofluorescence present in their sections or who wish to observe the plant anatomy.

#### Handling the plant material when sectioned

For the sections embedded in resin, using 10 well-slides present the advantage of having a relatively large amount of sequential sections per slide. To label the samples sectioned with a microtome and fixed on a slide coated with polylysine, we first isolate selected sections by placing a spacer on the slide or by marking a circle around the sections with a hydrophobic pen (e.g., Super PAP pen). For the immunolabeling of plant samples sectioned with a vibratome, we usually use cell culture plates with a flat bottom or centrifuge tubes and always incubate the sections in suspension with gentle agitation. It is important to note that extra care must be considered when manipulating samples in solution as the plant material can easily be damaged.

#### The immunolabeling procedure

To block non-specific binding, the sections of plant material are incubated with a fresh solution of phosphate-buffered saline (PBS, pH 7.2) containing a blocking agent (BA-PBS) (for example: 5% (m/v) milk protein or 3% (w/v) Bovine Serum Albumin) for 30 min at RT. Following a wash in PBS (5 min for the samples in suspension), the sections are incubated for at least 45 min at RT with a primary antibody that is directed to a plant cell wall epitope and that is diluted in BA-PBS. After extensive washes with PBS (three times 5 min for the samples in suspension), samples are incubated for at least 45 min at RT in the dark with a secondary antibody (e.g., anti-rat IgG or anti-mouse IgG) coupled to a fluorochrome. The antibody is diluted in BA-PBS. We do not recommend exceeding 6 h of incubation with the primary and the secondary antibody as the blocking agent can start to stick to the samples. From our experience, the ideal time of incubation is around 1 h. The samples are extensively washed and stained for 1 min at RT in the dark with either 0.1 M calcofluor (to observe the plant anatomy under UV wavelength) or with 0.05% (m/v) toluidine blue diluted in distilled water (to reduce the primary fluorescence and observe the plant anatomy in bright field). The sections are extensively washed one more time, disposed on a slide if necessary and soaked in an anti-fade reagent, such as citifluor AF1, to preserve the fluorochrome. Once soaked in a solvent, the slides are covered with a cover slip and ready for inspection under microscope. The immunolabelings of plant materials must be accompanied by at least one negative control. Sections incubated without primary antibody will allow the visualization of natural primary fluorescence present in the plant material.

## Results and discussion

### Embedding and sectioning of plant material

Immunolabeling of plant materials requires the use of high-quality sections and obtaining that is often not trivial. The importance of good sections is a point that is often overlooked. The quality and the quantity of samples sectioned and prepared for immunomicroscopy depend on the plant material and rely on the method of embedding and the corresponding mode of sectioning. Three techniques have been proven to deliver excellent materials suitable for immunolabeling: non-embedded samples sectioned with a vibratome, wax-embedded samples sectioned with a microtome, or resin-embedded samples sectioned with an ultramicrotome.

#### Vibratome

Of the three techniques, the sample preparation with a vibratome is the easiest to master. The vibratome has a user-friendly interface and it requires little skills to learn how to use it. The plant material is placed on a specimen holder that faces a knife holder. Speed and frequency are applied to the knife holder to move the blade toward the specimen and cut it. As the sample preparation does not require infiltrating the samples with a resin or paraffin, the structure of the samples is well-maintained and the procedure is extremely fast. The preparation and sectioning of plant material with a vibratome can be done within a few hours. Plant material can be directly mounted on the surface of a specimen holder by using cyanoacrylate glue. However, depending of the nature of the samples, coating agents may first be required for sample preparation. Common coating agents are Styrofoam, carrot dices, cork and agarose (Sallee and Russell, 1993; Ruzin, 1999). By comparison with the other methods and in absence of automation, using a vibratome is ideal to prepare large specimen for optical microscopy especially when it comes to reproducibility. As illustrated in Supplemental data S1, a relatively large number of biological replicates can be loaded on the specimen holder. For example, up to 20 stems of *A. thaliana* can be embedded in a single block of agarose to obtain transverse sections. However, for practical reasons, we do not add more than 11 stems on our specimen holder. Theoretically, a vibratome can section samples in a range of 10–200 μm using either disposable razor blades made of steel or a sharper knife such as a sapphire knife. For plant material and by using disposable steel blades, we have determined that the optimal thickness to obtain a good proportion of excellent sections was usually 60 μm. Once they are cut, the sections are collected in a buffer solution or in ultra-pure water. The sections obtained with a vibratome are not attached to a support. This can be advantageous for some applications; nevertheless, it also means that, for immunomicroscopy, these vibratome-cut sections are more delicate to manipulate than sections obtained with a microtome or an ultramicrotome. The sections cut with a vibratome can be stored at 4°C for a short period of time.

#### Microtome

Paraffin wax is a commonly used embedding medium, suitable for histochemical, immunolabeling and *in situ*-hybridization studies. Sections are prepared using a microtome. Regardless of whether they are manual or automated, microtomes work in a manner that is comparable to that of a vibratome. The major distinction is that, on a microtome, the specimen moves toward the knife and not the opposite. Using a microtome requires more skills and practice than using a vibratome and it is often more difficult to obtain good sections of plant material of large dimensions. The sample preparation for microscopy analysis takes a few days. The main steps consist in (i) dehydrating the fixed samples in a gradient series of incubation in aqueous alcohol, (ii) infiltrating the water-free samples with alkanes, commonly paraffin or Steedman's wax—it is to note that Steedman's wax embedding has been reported to be more suitable for immunomicroscopy than paraffin (Vitha et al., 2000), (iii) molding the samples in a block of wax or paraffin, and (iv) cutting the samples in ribbons using a sharp stainless steel blade that can be either disposable or reusable. (v) The ribbons are then disposed on a slide coated with an adhesive chemical, e.g., polylysine, and a drop of ultra-pure water is poured on the top of the slide to initiate the adhesion of the samples to the slide. (vi) Once fully dried, the alkanes are progressively dissolved by incubating the slide in a declining gradient of aqueous alcohol that is followed by washes in clean deionized water. The dried slides can then be stored for years at room temperature. Likewise, the blocks of wax-embedded samples can be stored for a long period of time if kept at cool or ambient temperature (no more than 25°C) with a moderate level of humidity. Recently, Francoz et al. have demonstrated that microtome-sectioning can be used to analyze populations of specimens within a realistic time frame. They have indeed successfully developed mid-throughput assays for *in situ* RNA hybridization of *A. thaliana* seeds where hundreds of siliques and, thus, thousands of seeds, were loaded in blocks of paraffin prior to sequential sectioning (Francoz et al., 2016). Nevertheless, to date, the number of biological replicates per block of wax is in general lower than what can be cut with a vibratome (see Supplemental data S1). For transverse stem sections of *A. thaliana*, for example, we usually position no more than 6 samples per block. Although sections of 1–100 μm can be obtained with a microtome, it has often been recommended to section plant material at 12 μm (Herve et al., 2011). Using a microtome is a rapid and convenient option when relatively thin sections are needed or when the samples cannot be cut with a vibratome, for example, due to the size or stiffness of the material. By comparison with the sections obtained with a vibratome, the thickness of the sections obtained with a microtome or an ultramicrotome is much more suitable for sequential analysis of plant material or co-localization studies.

#### Ultramicrotome

Resin-embedding and ultramicrotome-sectioning are commonly used for immunocytochemistry especially when both light and electron transmission microscopy are envisioned. Like the protocol for wax-embedding, the preparation of samples requires to first dehydrate the samples in a gradient series of incubation in aqueous alcohol. The samples are then infiltrated with a resin. Acrylic resins are preferred to epoxy resins for immunolabeling studies. The hydrophobic nature of epoxy resins and the heat curing effects are generally detrimental to antigen preservation resulting in a low immunolabeling efficiency (De Paul et al., 2012). The LR White resin is the most commonly used resin for immunomicroscopy (Yeung and Huang, 2015) and, for plant material, we recommend the hard grade LR White resin. This hydrophilic acrylic resin polymerizes via a free radical mechanism. It is thus important to exclude oxygen during the step of polymerization that follows the infiltration. For this purpose, Bowling and Vaughn (2008) have developed a simple method that relies on the use of dental wax and that enables to visualize the end of the polymerization. To limit any loss of antigenicity, these authors advise to polymerize LR White at 55°C and to reduce the time of polymerization to its minimum (Bowling and Vaughn, 2008). When the polymerization is complete, the block can be either cut with glass or diamond knifes or stored in dry conditions for years. Similarly to microtome-sectioning, the sections are cut in ribbons. The ribbons are collected from a tank filled with ultra-pure water and disposed on a slide coated with an adhesive. For resin-embedded sections, we use Vectabond™ reagent as an adhesive and 8- to 12-well slides. To preserve the antigenicity of the sections, the slides are dried overnight at room temperature rather than being heated. By contrast with wax-embedding, the resin is not removed from the sections and, once dried, the slides are ready for analysis or can be stored for years at ambient temperature. Sectioning with an ultramicrotome requires practices and accomplished skills and the technique is demanding. However, it offers several advantages. To our knowledge, any plant material, regardless of its nature and composition, can be cut with an ultramicrotome once it is embedded in resin. We have observed, for example, that, when preparing transverse sections of mature rice stem, only a few sections could be cut with a vibratome or a microtome before damaging the blades. By contrast, a diamond knife does not suffer from the high level of silica contained in rice stems and we have successfully obtained excellent sequential sections with an ultramicrotome. Opting for ultramicrotome-sectioning is particularly convenient when working with small specimens or hard tissues. Of the three techniques, ultramicrotome-sectioning of resin-embedded plant material is the only option to analyze samples by both light and electron microscopy. The sections obtained with an ultramicrotome can range from 0.01 to 2 μm. For immunomicroscopy with a light microscope, we usually section our plant material at 0.5 or 1 μm. Once the correct protocol is applied, the ultramicrotome-sectioning allows to get thin sections that provides a definition and a quality under the microscope that cannot be obtained with the two other methods. The downside is that it is time-demanding to obtain the perfect section as the protocol is sample-dependent and that manipulating resin-infiltrated samples is delicate. Furthermore, by comparison with wax-embedding, the samples are more easily altered by the resin infiltration. Another limiting aspect of ultramicrotome-sectioning is the number of samples that can be prepared at a given time. Although the steps of dehydration and infiltration can be automated, only one sample can be prepared per block and only one sample can be cut at a time, and therefore the number of replicates must be minimized. Of the three methods, the preparation of samples through ultramicrotome-sectioning is the longest. Shorter protocols using, for example, microwave methods (Yeung and Huang, 2015) have been reported where the procedure can be performed within a few days. However, in our experience, these fast protocols are not ideal for immunomicroscopy of plant cell walls and the protocol that fits best for such application requires about a week.

### Impact of the sample preparation on cell wall staining and immunolabeling with monoclonal antibodies directed to cell wall polysaccharides

As mentioned above, resin, wax and agarose embedding as well as their associated methods of sectioning determine the thickness of the sections. This has a direct impact on the quality of the sections. To illustrate the impact of sample preparation on the quality of the sections, the intensity of fluorescence, the antigenicity of the samples and the effect of chemical and enzymatic treatments, we use equivalent stem material from *A. thaliana*. Unless notified, 1 cm-long stem segments were collected from 15 cm distal of the rosette from inflorescence stems with an average height of 30 cm. By contrast with the base of the stems or the apices, the segments collected from this region were easy to handle what facilitated the comparison in between the three methods presented here.

### Section quality and tissue integrity

**Figure 1:**
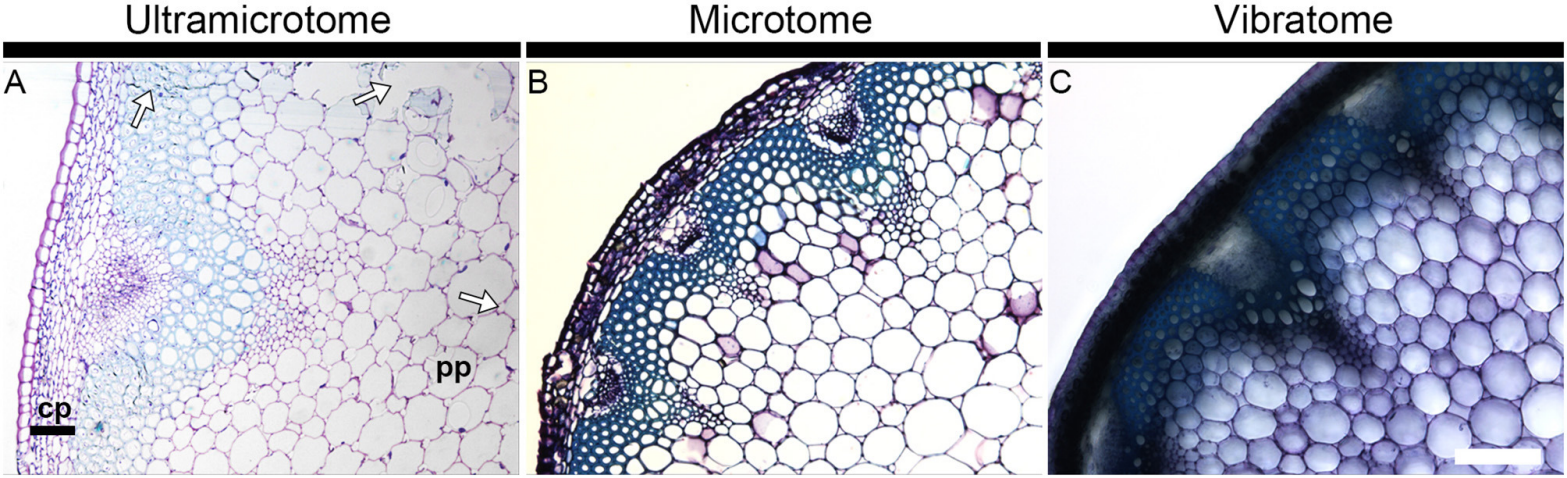
Section quality and tissue integrity of plant material depend of the sample preparation. The micrographs show transverse *A. thaliana* stem sections obtained with an ultramicrotome **(A)**, a microtome **(B)** and a vibratome **(C)** and stained with toluidine blue. The thickness of the sections is 0.5, 12, and 60 μm, respectively. The staining with toluidine blue highlights how the image definition depends on the thickness of the section and how tissue integrity depends on the method of sectioning and its corresponding mode of embedding. The arrows in micrograph A highlight regions where the tissue integrity has been affected by the procedure of resin embedding. Note that equivalent alterations can occur when sections are embedded in wax. cp, cortical parenchyma; pp, pith parenchyma. Scale bar: 200 μm.

Figure 1 illustrates how the section quality and the tissue integrity rely on sample preparation. It shows bright field photographs of transverse sections of *A. thaliana* stems stained with toluidine blue, a reagent routinely used in microscopy to stain cell walls. Whereas the section obtained with an ultramicrotome (Figure 1A) exemplifies several quality issues, the other micrographs are representative of the optimal quality of sections that can usually be expected when sectioning plant material with either a microtome (Figure 1B) or a vibratome (Figure 1C). The artifacts shown in Figure 1A are representative of the tissue alterations caused by a procedure of resin-embedding that was not best suited for this specific stem segment - we used the protocol termed “soft” in our Material and Methods whereas the “hard” protocol would have been more appropriate. A close-up on the cortical parenchyma (cp) in the micrographs demonstrates the limits that results from the thickness of the sections. In the section embedded in resin and cut with an ultramicrotome (0.5 μm thick), each individual cell can be visualized (Figure 1A). By contrast, the cells present in this tissue are barely distinguishable in 12 μm-thick sections obtained by wax-embedding and microtome-sectioning (Figure 1B). The cortical parenchyma cells cannot be dissociated from each other in the 60 μm-thick section coated in agarose and cut with a vibratome (Figure 1C). The definition and the visibility of individual cells declines from the sections prepared with an ultramicrotome to the sections prepared with a microtome to the sections prepared with a vibratome. On the other hand, for samples that contain cell layers with distinct mechanical properties, soft tissues such as the pith parenchyma cells (pp) are better preserved when sectioned with a vibratome than with a microtome or an ultramicrotome. This is not only a consequence of the large thickness of the section—the integrity and the structure of the tissues are less weakened/better maintained in a 60 μm thick section than in 12 or 0. 5 μm thick sections—but also results from the absence in the vibratome procedure of alcohol dehydration and infiltration with wax or resin. These two steps induce changes of pressure and alter the intrinsic properties of cells. When the cells are not able to sustain these changes, it can cause excessive hardening, tissue shrinkage or cell distortion (Westra et al., 2003). In soft tissues, it may be reflected by collapsed areas. Examples of these tissue alterations are highlighted by arrows in Figure 1A. It is to note that equivalent regions of torn tissues, as seen in Figure 1A, may also occur when the samples are not carefully handled. Yet, we have observed that, by comparison with sections embedded in resin or wax, such artifacts rarely occur when the sections are cut with a vibratome. Adapting the gradient of alcohol, for example, by empirical addition or subtraction of steps or by modifying the time of incubation, can prevent or limit the damages caused by alcohol dehydration and infiltration with wax or resin (see Material and Methods). However, because the resistance to dehydration and infiltration varies between tissues, there is not always an ideal protocol for sectioning heterogeneous plant material such as mature *A. thaliana* stem, and it is necessary to find a compromise to deal with the fragility of soft tissues vs. the strong resistance to infiltration of hard tissues. When comparing cell wall mutants or transformants with a wild-type or non-transformed plant, observing an altered fragility of samples to the procedure of wax or resin embedding can be a useful indication for cell wall alterations as cell integrity relies on the walls. Moreover, as the blades apply a constant shear force while moving toward the materials, significant alterations in the mechanical properties can easily be detected when sectioning the samples with a vibratome or a microtome.

### Primary fluorescence

**Figure 2:**
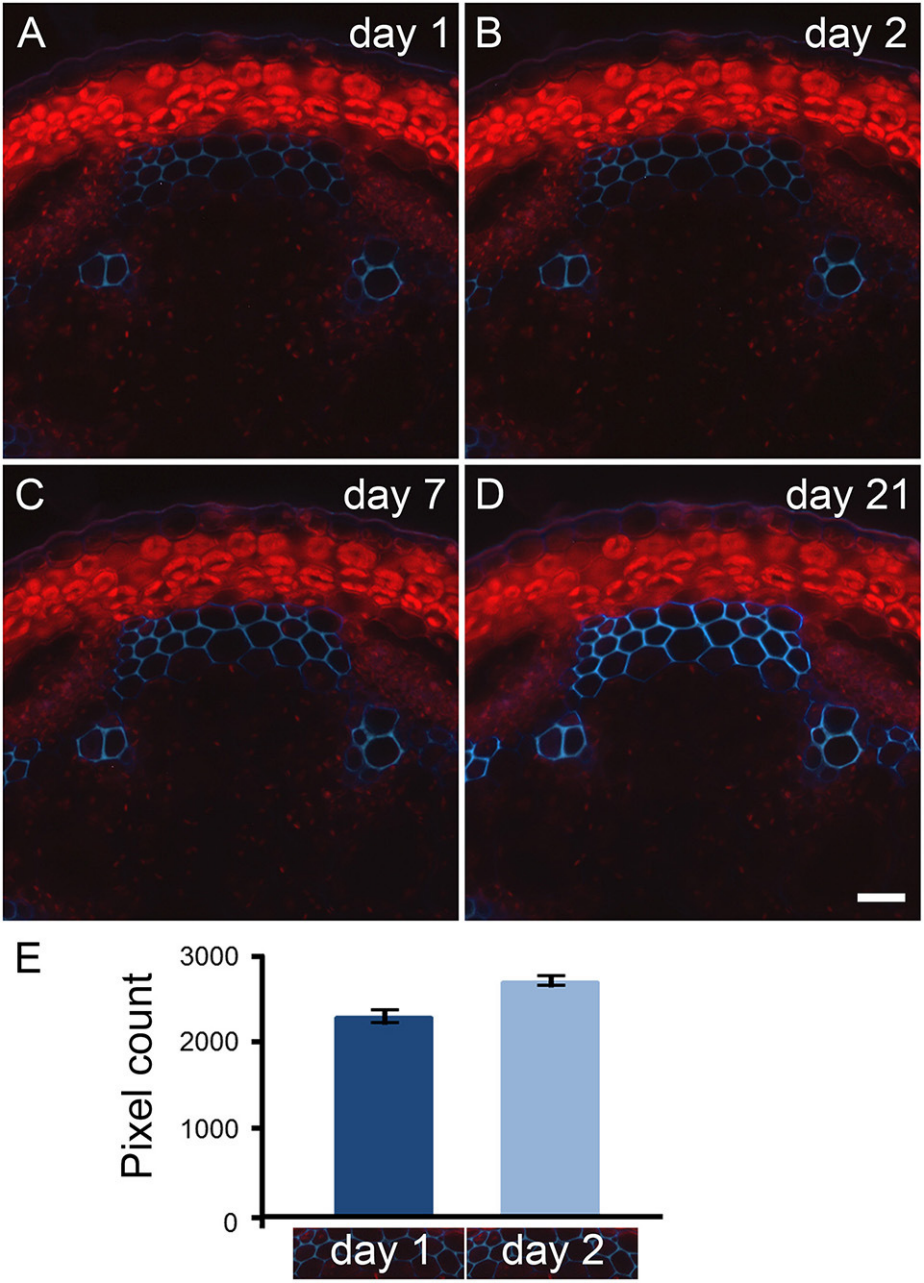
Primary fluorescence increases over time when transverse stem sections are stored in paraformaldehyde. Transverse sections of *A. thaliana* stems were stored in 4% paraformaldehyde for 21 days. The same sections cut with a vibratome were observed with UV excitation after 1 **(A)**, 2 **(B)**, 7 **(C)**, and 21 **(D)** days of incubation in paraformaldehyde. Using a long pass emission filter allows the visualization of primary fluorescence produced by the interfascicular fibers (blue color) and by the plastids located in the cortical parenchyma (red color). Comparison between micrographs **(A–D)** shows the increase of autofluorescence that occurs over time when the stem sections are stored in paraformaldehyde. Scale bar for **(A–D)**: 30 μm. **(E)**: The measurement of light blue color pixels from the same interfascicular regions of stem sections incubated for 1 and 2 days in 4% paraformaldehyde indicates that the increase of autofluorescence already occurs after 2 days of incubation. The pixel measurement was carried out using ImageJ following the procedure detailed in Supplemental data S2. The micrographs underneath the graph represent the region selected for pixel measurement. For this example, the measurement was done in triplicate. Error bars: SD.

Natural fluorescence of plant material, referred as primary fluorescence or autofluorescence, is abundantly detected where phenolic compounds are accumulated. Typically, primary fluorescence is observed in wax and cutin at the surface of the epidermis, in the sub-epidermal layers from plastids (see Figure 2), suberin or collenchyma (Leroux, 2012), and in cell types that possess lignin and/or feruloylated components. Through our observations of fern, dicot and monocot specimens, we have noticed that the fixation of plant material with paraformaldehyde leads to an increase of primary fluorescence over time. This is illustrated in Figure 2 with a transverse stem section of *A. thaliana*. Stem segments of 1 cm were collected from a region 5 cm distal of the rosette from inflorescence stems with an average height of 10 cm. The sections were cut transversely with a vibratome, and stored in 4% paraformaldehyde for 21 days. The same sections were observed with UV excitation [excitation filter: band pass (340–380 nm), emission filter: long pass (425 nm)] after 1, 2, 7, and 21 days of incubation in paraformaldehyde and photographed with the exact same parameters (Figures 2A–D). Day 1 corresponds to an incubation time of about 16 h (see Material and Methods). A subtle increase of autofluorescence in the interfascicular fibers was already recorded by pixel measurement after 2 days of incubation in the fixative solution (Figure 2E). After 7 days of incubation in paraformaldehyde, the increase of primary fluorescence in the interfascicular fibers was clearly visible. The intensity of autofluorescence keeps increasing up to 21 days of incubation. To ensure that the effect was due to paraformaldehyde and not its buffer solution, we have tested two solvents, a PEM buffer (pH 6.9) and a phosphate buffer (pH 7.2), in which we have dissolved the fixative reagent. We obtained equivalent result. In conclusion, to limit the intensity of primary fluorescence, it is recommended to store plant material in the fixative solution for the shortest amount of time.

**Figure 3:**
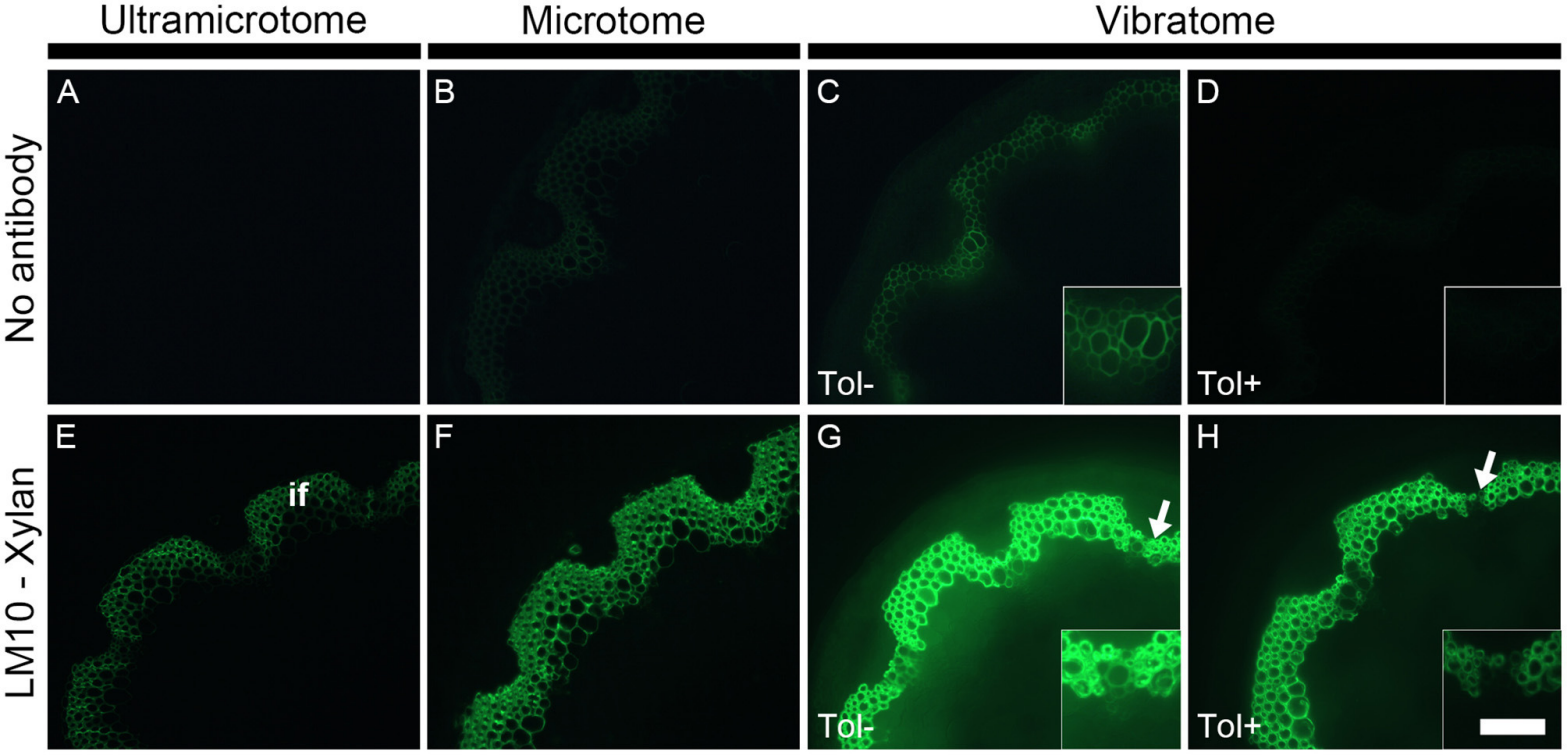
The detection of primary fluorescence and indirect immunolabeling relies on the preparation of plant samples. Sections cut with either an ultramicrotome **(A,E)**, a microtome **(B,F)** or a vibratome **(C,D,G,H)** were obtained from equivalent stem regions (ca. 15 cm distal from the rosette of stems with an average height of 30 cm) and photographed by applying the exact same parameters (e.g., same time of exposure). Micrographs **(A–D)** show the primary fluorescence of equivalent transverse stem sections of *A. thaliana* under blue excitation. This fluorescence is naturally emitted, for example, by phenolic compounds such as the lignin present in the interfascicular fibers (if). Micrographs **(E–H)** show the indirect immunolabeling of xylan, a cell wall polysaccharide recognized by the LM10 monoclonal antibody. The primary antibody binding is revealed with a secondary anti-rat monoclonal antibody coupled with a FITC fluorochrome. The intensity of both primary fluorescence and the artificial fluorescence resulting from the immunolabeling are proportional to the thickness of the sections. As shown here, both type of fluorescence increase with section thickness. The sections obtained with a vibratome were used to demonstrate the effect of toluidine blue when the sections are excited under blue light. When overlapping with the artificial fluorescence, the primary fluorescence in absence of toluidine blue (Tol −) (**C,G** and their insets) can impede the interpretation of immunolabeling. A staining with toluidine blue (Tol +) **(D,H)** drastically reduces the autofluorescence (**C** vs. **D**) with, in this example, a minimal loss of epitope detection (**G** vs. **H**, insets). The arrows in micrographs **(G,H)** highlight regions where the LM10 antibody bound weakly to cell walls when the sections were stained after immunolabeling. Scale bar: for the large micrographs: 100 μm, for the insets: 50 μm.

Due to the high diversity of fluorescent compounds present in plants, a relatively strong autofluorescence is usually visible throughout the entire spectrum of wavelengths used in fluorescent microscopy. This is a major concern when analyzing samples by immunomicroscopy since primary fluorescence overlaps with the emission of fluorescence produced by the fluorochromes used to detect cell wall epitopes (see Figure 3). In some cases, it can be extremely difficult to visualize the presence of cell wall polymers because of primary fluorescence. As demonstrated in Figure 3, the intensity of fluorescence depends of how the samples are prepared (Figures 3A–C). Under the same conditions—which include the same emission and excitation wavelength [Excitation filter: Band pass (470–510 nm), Emission filter: Band pass (525–575 nm)] and the same time of exposure for capturing the image - the intensity of autofluorescence increases from the thinnest section (Figure 3A) to the thickest section (Figure 3C). Resin-embedded samples usually exhibit a low level of autofluorescence when observing the sample under the microscope through the eye-pieces. Consequently, no fluorescence is detected when photographing the sample with a short time of exposure (Figure 3A). By contrast, when applying the same parameters to the thicker wax-embedded samples, primary fluorescence is visible in the ring of interfascicular fibers and the xylem cells of the wax-embedded stems (Figure 3B). These cell types strongly autofluoresce in sections that have not been infiltrated with wax or resin and cut to a thickness of 60 μm with a vibratome (Figure 3C). The thickness of the samples and the embedding procedures are responsible for these differences of fluorescence intensity. For the same surface, the thicker a plant sample is, the more it contains fluorescent compounds and thus the higher the intensity of fluorescence is. Moreover, the dehydration and infiltration steps performed during the resin and wax embedding lead to some loss of naturally fluorescent compounds.

Changes in primary fluorescence will be more easily perceived in thick samples than in relatively or semi-thin sections where the autofluorescence can sometimes barely be visible. Therefore, the use of vibratome is adequate for studying phenolic compounds such as lignin by fluorescence microscopy. By contrast, resin-embedded sections are preferable when the presence of primary fluorescence is not desired (see Supplemental data S3). There are several approaches to reduce the primary fluorescence when its presence is an issue. Using a band pass emission filter (as done in Figure 3) instead of a long pass filter (see Figure 2) and/or observing the samples with red light excitation (530–595 nm) will narrow the spectrum of fluorescence observed by microscopy. The primary fluorescence can be quenched by exposing the samples to intense light prior to labeling. However, photobleaching damages the samples and the technique is not convenient for immunolabeling of plant cell walls. It is possible to bypass the autofluorescence by carrying out immunogold labeling followed by silver enhancement and fuchsin couterstaining with observation of the samples by epi-polarized light microscope (Bush and McCann, 1999). Yet, as it is easier, cheaper and faster, we stain the sections with toluidine blue. This dye considerably reduces the detection of primary fluorescence under blue excitation (Excitation: 450–495 nm) (Biggs, 1985; Leroux et al., 2015). This is demonstrated in Figure 3 where a strong level of autofluorescence is observed in an untreated stem section cut with a vibratome (Figure 3C, Tol−) but drastically diminished in an equivalent section stained with toluidine blue (Figure 3D, Tol+). As toluidine blue binds to lignin and feruloylated polysaccharides (Smith and McCully, 1978), the quenching of autofluorescence occurs regardless of how the plant material is prepared. However, it is recommended to analyze the toluidine blue-stained sections under blue excitation using a band pass filter as emission filter. Toluidine blue possesses a phenolic ring that fluoresces when excited from ca. 560 nm (Ilanchelian et al., 2000).

### Antibody fluorescence

**Figure 4:**
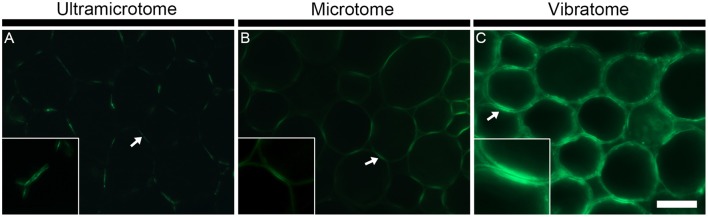
The sample preparation affects the antigenicity and/or abundance of cell wall epitopes. The figure shows representative indirect immunolabeling of the LM16 pectic arabinan epitope in pith parenchyma cells from transverse stem sections of *A. thaliana*. **(A)** Section embedded in LR White resin and cut with an ultramicrotome. **(B)** Section embedded in Steedman's wax and cut with a microtome. **(C)** Section coated in agarose and cut with a vibratome. The binding of the LM16 antibody varies depending on the sample preparation. The arrows indicate the region of adhered cell walls where the LM16 epitope is abundantly detected. The insets at 2x scale highlight that a reduced antigenicity of the sample can provide better details on the fine localization of the epitopes. In the insets, the epitope is seen to be present in the primary cell walls and absent from the middle lamella. The micrographs were taken with different times of exposure that were, for each method of sample preparation, determined based on a signal to noise ratio (negative controls are shown in Supplemental data S4). The shortest and longest times of exposure were taken for the sections cut with a vibratome and an ultramicrotome, respectively. Scale bar for the large micrographs: 70 μm, for the insets: 35 μm.

**Figure 5:**
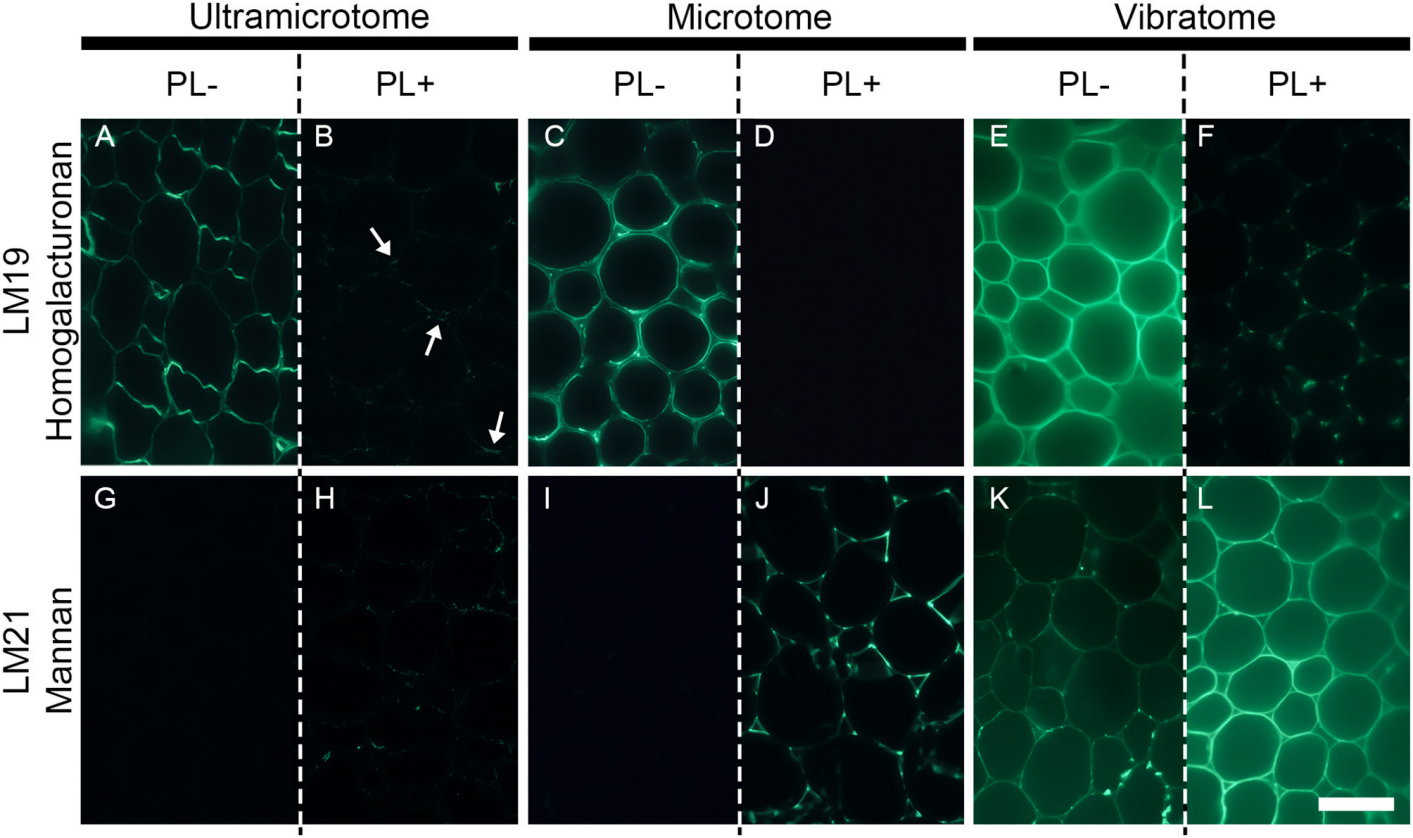
The efficiency of enzymatic pre-treatment depends of the sample preparation. The figure shows the impact of pectate lyase pretreatment on indirect immunolabeling of the LM19 homogalacturonan and the LM21 mannan epitopes in pith parenchyma cells of transverse *A. thaliana* stem sections. The presence of the LM19 homogalacturonan epitope is ubiquitous in the walls of pith parenchyma cells and the degradation of homogalacturonan with pectate lyase results in a severe reduction of LM19 detection and an increased detection of the LM21 mannan epitope. The immunolabeling obtained after enzymatic pretreatment showed nuances of enzymatic efficiency between the different methods of sample preparation. **(A,B,G,H)** Section embedded in resin and cut with an ultramicrotome. **(C,D,I,J)**: Sections embedded in wax and cut with a microtome. **(E,F,K,L)**: Sections coated in agarose and cut with a vibratome. **(A–F)** Representative sections immunolabeled with the LM19 homogalacturonan antibody. **(G–L)**: Representative sections immunolabeled with the LM21 mannan antibody. **(A,C,E,G,I,K)** show sections incubated for 2 h with phosphate buffer (pH 7.2) (PL-) prior to immunolabeling. **(B,D,F,H,J,L)** show sections incubated for 2 h with pectate lyase in a CAPS buffer (pH 10) (PL+) prior to immunolabeling. The parameters used to capture these micrographs were identical between the pre-treatments. However, the time of exposure differed for both antibodies and for each method of sample preparation. The shortest and longest times of exposure were taken for the sections cut with a vibratome and an ultramicrotome, respectively. The arrows in B highlight regions where residual traces of the LM19 epitopes are detected after pectate lyase pre-treatment. Scale bar: 70 μm.

As illustrated in Figure 3 with LM10, an antibody directed to hemicellulosic xylan, the intensity of fluorescence resulting from immunolabeling depends on the method of sample preparation (Figures 3E–G). Under the same conditions, the occurrence of the LM10 epitope in the interfascicular fibers and xylem cells is weakly detected in the transverse stem sectioned with an ultramicrotome (Figure 3E), the intensity of fluorescence is higher in the section prepared with a microtome (Figure 3G) and the fluorescent signal is saturated in the sections cut with a vibratome (Figure 3F). In our assay, the procedures of embedding and the sample thickness are the only two parameters that differ between the three types of sections. The difference of fluorescence detection likely relies on both parameters. In resin-embedded sections, the presence of the LR White resin prevents antibodies from penetrating into the sections so only the epitopes that are accessible at the surface can be detected. Sections embedded in wax or coated in agarose are not impacted by the presence of the embedding agent. Consequently, in addition to the section surface (X-Y-axis), the antibodies may also access the cell walls present in the inner surface (Z-axis) of these sections. It is reasonable to think that, in sections embedded in wax or coated in agarose, the antibody binding intensity increases with the thickness of the sections since the surface area, and thus the amount of epitopes accessible to the antibodies, are directly proportional. In addition to the dimensional aspect of the section, the difference in the intensity of fluorescence detected may also be explained, at least partially, by loss of accessible cell wall epitopes that occurs during the steps of dehydration and infiltration (see Figures 4, 5 and the section “Immunolocalization”).

**Figure 6:**
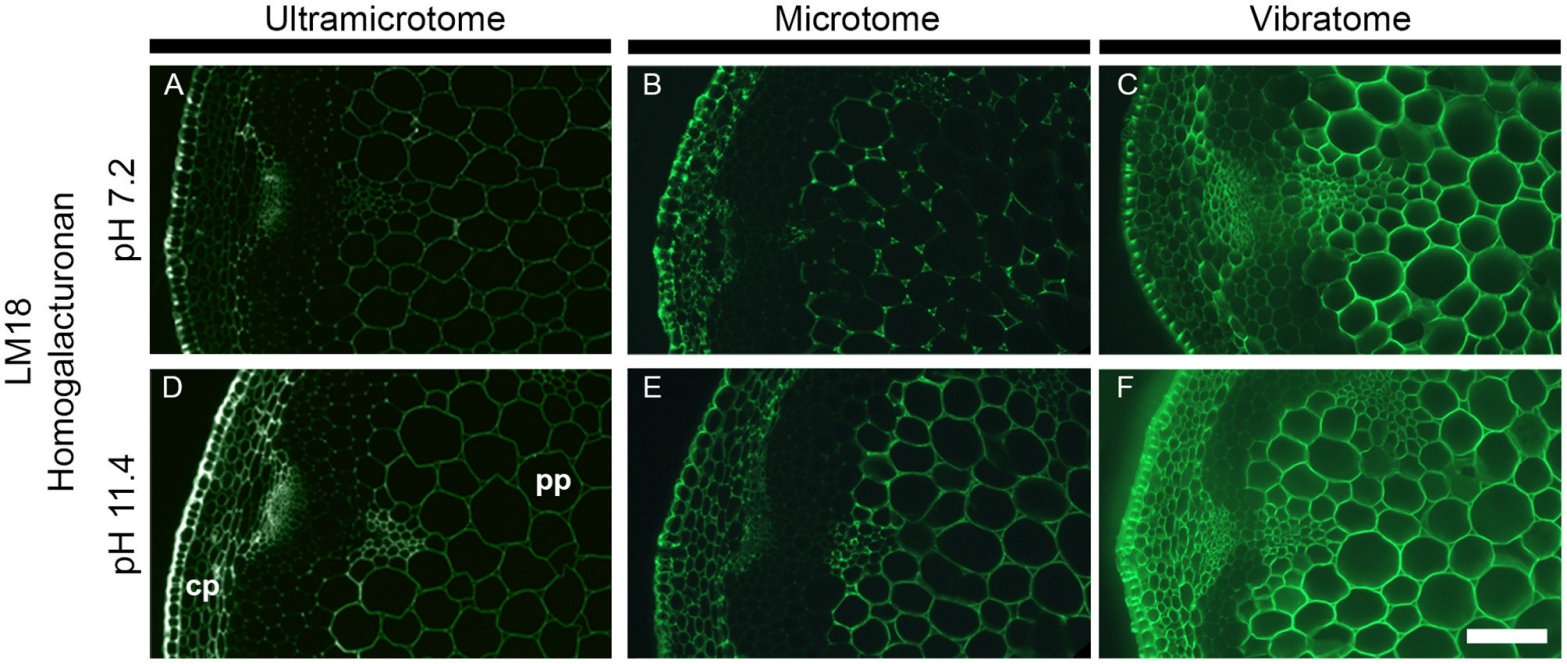
Impact of the sample preparation on indirect immunolabeling after alkaline pre-treatment. Transverse stem sections of *A. thaliana* have been incubated for 2 h with either phosphate buffer (pH 7.2) or sodium carbonate (Na_2_CO_3_) (pH 11.4) prior indirect immunolabeling with the LM18 antibody. As represented in this figure, the alkaline pre-treatment results in an increase of LM18 detection regardless of the how the samples were prepared. **(A,D)** Section embedded in resin and cut with an ultramicrotome. **(B,E)** Sections embedded in wax and cut with a microtome. **(C,F)** Sections coated in agarose and cut with a vibratome. **(A–C)** were incubated with phosphate buffer (pH 7.2) prior to immunolabeling with the LM18 homogalacturonan antibody. **(D–F)** were pre-treated with Na_2_CO_3_ (pH 11.4) prior to immunolabeling with the LM18 antibody. The parameters used to capture these micrographs were identical between the pre-treatments. However, the time of exposure differed for each method of sample preparation. cp, cortical parenchyma; pp, pith parenchyma. Scale bar: 70 μm.

To avoid misleading the readers with this comparative assay, we must emphasize that what is important when performing immunolabeling is to ensure that the same times of exposure are applied to both immune- and control sections, regardless of which method is used (see, for example, Figure 6). In Figure 3, the same time of exposure was used for each micrograph to illustrate the difference of fluorescence intensity that occurs in between the three methods of sample preparation. However, since the resin-embedded sections show little if any primary fluorescence (Figure 3A), it is not problematic to apply longer exposure times (see Supplemental data S3) to achieve images that are comparable in brightness to the microtome or the vibratome sections shown here in Figures 3F,G, respectively. Likewise, for the sections cut with a vibratome, shorter time of exposure can be used to reduce the primary fluorescence and avoid a saturation of the fluorescent signal (Supplemental data S3).

When the sections are stained with toluidine blue, the accumulation of green fluorescence emitted from both the plant material and the fluorescent probe is diminished. As a result, the signal detected under the microscope is less intense in sections stained with toluidine blue (Figure 3H, Tol+) than in the sections that are not stained (Figure 3F, Tol−). The post-immunolabeling stain with toluidine blue greatly facilitates the distinction between the fluorescence produced by the labeling from the primary fluorescence (Figures 3C,F vs. Figures 3D,H). However, this easy method of autofluorescence quenching may also lead to a loss of information. For instance, the occurrence of cell types weakly detected in unstained sections may sometime be barely or no longer visible in sections stained with the dye. Such loss of detection can be observed when comparing micrograph Figures 3G,H in the regions highlighted by arrows and magnified in the insets. The reduction of detection is associated with the binding of toluidine blue to lignin and feruloylated polysaccharides and varies with the targeted epitopes as well as the source of plant material. Prior to using it routinely, it is thus important to evaluate the effect of a post-immunolabeling stain with toluidine blue and to determine how it can impact the results.

Most fluorochromes attached to secondary and tertiary antibodies are prone to photobleaching. We have particularly noticed this when using fluorescein isothiocyanate (FITC). To bypass the sensitivity of the fluorophore, immunolabeled samples can be soaked in protective solutions that prevent a rapid photobleaching. We routinely use Citifluor AF1, a glycerol phosphate buffered solution containing antifadent, and have observed that, when using FITC, the immunofluorescence was still well present in samples stored for over 6 months at 4°C. Alternatively, stable and robust fluorophores such as Alexa Fluors can be used in absence of antifadent solution (e.g., Ralet et al., 2010; Phan et al., 2016) as they can undergo long light exposure before photobleaching under light epifluorescence microscopy.

### Immunolocalization

The technique of embedding can influence how epitopes are detected in plant material, not only in term of fluorescence intensity but also in term of localization. How the methods of sample preparation impact on the immunolocalization varies with the epitope. Usually, immunolabelings show none or subtle differences of epitope occurrence between sections embedded in acrylic resin or wax or coated in agarose. For instance, in the transverse *A. thaliana* stem sections labeled with the LM10 antibody, there is a difference of intensity of fluorescence between the sections cut with an ultramicrotome, a microtome or a vibratome but no change in epitope occurrence (Figures 3E–G). The LM19 homogalacturonan epitope detection in the parenchyma cells of *A. thaliana* stem shown in Figure 5 (Micrographs A, C, E) is a second example. Nevertheless, the loss of detection can sometimes be drastic for other epitopes. This is exemplified in Figure 4 with the LM16 arabinan antibody. As previously reported (Verhertbruggen et al., 2009b), the LM16 epitope is strongly detected in the adhered region of parenchyma cell walls, with rare patches detected at cell corners (Figure 4A). Embedding in wax (Figure 4B) results in a more abundant detection of the LM16 epitope than what is observed in the resin section. Albeit weak, the epitope occurrence is visible all around most cell corners. The absence of LM16 epitope from the middle lamella can easily be observed in the wax-embedded section (see inset in micrograph B). In the non-infiltrated sections, the arabinan epitope is consistently detected all around the cells and the overall intensity of fluorescence is high (Figure 4C). Overall, the abundant detection of the LM16 arabinan epitope in the adhered walls of the cells and its absence from the middle lamella are visible regardless of the method of sample preparation used. It is to note that, when looking through the eye-pieces, these observations are less easily noticed in sections cut with a vibratome than in sections cut with an ultramicrotome or a microtome. In this Figure, we have used different times of exposure to highlight in each type of sections where the LM16 epitope is immunolocalized. Severe loss of detection that is related to the method of sample preparation has already been reported for the LM7 epitope (Willats et al., 2001) and can also be observed in Figure 5 for the LM21 mannan epitope. In absence of pre-treatment, the LM21 epitope can only be detected in the parenchyma cells of *A. thaliana* stem when the sections are not embedded in wax or resin (Figure 5, Micrographs G, I, K) and, thus, not subject to steps of dehydration and infiltration. If the thickness of the samples was the only parameter to affect its detection, the LM21 epitope would have been expected to still be visible in the microtome sections. This was neither observed here or by Marcus et al. (2010). Altogether, these results strongly suggest that the procedure of embedding can affect the preservation of certain epitopes. We do not know what exactly causes the loss of carbohydrate epitopes but it is possible that they are extracted either by water or by alcohol and altered by the changes of pH that occurs during the steps of alcohol dehydration and infiltration.

Bypassing dehydration and infiltration preserves the antigenicity of the sections and, consequently, sectioning plant material with a vibratome allows obtaining the best vision of what the real epitope occurrence *in muro* is. However, vibratome-sectioning is not the most convenient technique when the aim is to inspect the *in fine* occurrence of epitope. Extra precautions must be taken to ensure that what is seen does not come from artifacts resulting from the thickness of the sections. Resin-embedded sections present the advantages that they can be used both for light and electron microscopy. This is obviously of a great advantage when the aim is to gain knowledge on the ultrastructure of plant cell walls. However, for light microscopy, the loss of antigenicity and the cell wall alterations that can result from the embedding in resin or the presence of the resin itself are disadvantageous. The wax-embedded sections also endure damages during the steps of dehydration and infiltration but, as the sections are dewaxed, the accessibility to the epitopes is not directly affected by the presence of the polyester. We have often observed that the wax procedure provides a good definition for studying details in the cell walls with a light microscope (see Figures 4–6 as example).

### Alkaline pre-treatment

The combination of alkaline pre-treatment with immunolabeling can be used either to directly study the methyl- or acetyl-esterification of cell wall polysaccharides (as carried out, for example, by Verhertbruggen et al., 2009a; Koutaniemi et al., 2012). Furthermore, the alkaline pre-treatment can be used to remove ester groups present on cell wall polysaccharides to study other cell wall features by immunomicroscopy (Marcus et al., 2010). To demonstrate how the sample preparation impacts on alkaline pre-treatments, we have chosen the LM18 antibody, which recognizes a partially methyl-esterified homogalacturonan epitope, and pre-treated stem sections for 1 h with either a phosphate buffer (pH 7.2) or 0.1 M sodium carbonate (pH 11.4) (Figure 6). As shown in Verhertbruggen et al. (2009a), the detection of the LM18 epitope is enhanced by alkaline pre-treatment. Compared to sections pre-incubated for 1 h in a phosphate buffer, the epitope detection is higher in transverse stem sections pre-incubated in 0.1 M sodium carbonate (Figure 6A vs. Figure 6D, Figure 6B vs. Figure 6E, and Figure 6C vs. Figure 6F). This is clearly visible in the cortical parenchyma of the resin-embedded sections (Figures 6A,D). The difference in epitope detection in the pith parenchyma is more contrasted in the section cut with a microtome (Figure 6B vs. Figure 6E), than in the sections cut with an ultramicrotome (Figure 6A vs. Figure 6D) or a vibratome (Figure 6C vs. Figure 6F). This is particularly striking when comparing the sections through the eye-pieces. The intensity of fluorescence being already high in the untreated sections cut with a vibratome (Figure 6C), the increase of detection that occurs following an alkaline pre-treatment (Figure 6F) is harder to observe when comparing the samples under the microscope. Here, the variation of fluorescence intensity that occurs between the different tissues (cp vs. pp) is more visible in the ultramicrotome cut sections pre-treated with sodium carbonate than the other sections. However, from our experience, these variations, easily seen on images acquired with sufficient exposure time, are sometimes difficult to perceive through the eye-pieces. In addition to these observations, we have also observed that harsh pH pre-treatment (higher than pH 12) released the sections from the slides that were coated with Vectabond. If the results obtained after an alkaline pre-treatment are similar between the three procedures of sample preparation, ultramicrotome-sectioning and microtome-sectioning appear to be better options than vibratome-sectioning for this type of application.

### Enzymatic pre-treatment

Enzymatic pre-treatments are useful to study cell wall degrading-enzymes and their bioengineering (Zhang et al., 2014; Venditto et al., 2015), cell wall architecture and the function of cell wall polysaccharides (Marcus et al., 2008), and to understand how to fine-tune cell wall deconstruction (Hervé et al., 2010; Zhang et al., 2014). To demonstrate the effect of sample preparation on enzymatic pre-treatments, we have reproduced for each method the experiment performed by Marcus et al. (2010). Transverse stem sections of *A. thaliana* were pre-treated with 10 μg mL^−1^ of recombinant *Cellvibrio japonicus* pectate lyase (Megazyme, Bray, Ireland) diluted in 50 mM 3-(cyclohexylamino)pro-panesulfonic acid (CAPS), 2 mM CaCl_2_ buffer (pH 10) for 2 h at room temperature. The sections were then immunolabeled with either the LM19 homogalacturonan antibody or the LM21 mannan antibody. As shown in Marcus et al. (2010), the presence of the LM19 homogalacturonan epitope is ubiquitous in the walls of pith parenchyma cells and the degradation of homogalacturonan with pectate lyase results in a depleted detection of the LM19 epitope and an increased detection of the LM21 mannan epitope (Figure 5). Nonetheless, the immunolabelings show nuances between the different methods of sample preparation. As mentioned previously, the intensity of fluorescence increases, respectively, from the labeled sections cut with an ultramicrotome (Figure 5A), to the sections cut with a microtome (Figure 5C), to the sections cut with a vibratome (Figure 5E). Moreover, the efficiency of pectate lyase is affected by the method of sample preparation. In the resin-embedded section, residual traces of the LM19 epitope are still visible after the enzymatic pre-treatment (Figure 5B). The enzymatic treatment leads to a complete loss of the LM19 epitope detection in the wax-embedded sections (Figure 5D) while it reveals the presence of microdomains in the pith parenchyma cells sectioned with a vibratome (Figure 5F). Likewise, while the unmasking of the LM21 mannan epitope with pectate lyase is visible regardless of how the sections are prepared, the efficiency of the enzyme to unmask mannan varies with the method of embedding and sectioning. In the ultramicrotome-cut section, the LM21 epitope is not detected in absence of pre-treatment (Figure 5G) and only traces are visible after the enzymatic pre-treatment (Figure 5H). In the wax-embedded section, the occurrence of the LM21 epitope is undetected in the untreated sections (Figure 5I) and the pectic degradation unmasks its presence (Figure 5J). When they are cut with a vibratome, the occurrence of the LM21 epitope in the walls of pith parenchyma cells can already be observed in absence of enzymatic pre-treatment (Figure 5K), and the weak signal in the untreated sections is much higher after the removal of homogalacturonan (Figure 5L). Enzymatic pre-treatment on resin-embedded sections have delivered excellent results (Roland and Vian, 1981; Ruel and Joseleau, 1984; Luis et al., 2013; Chateigner-Boutin et al., 2014; Buffetto et al., 2015). For example, pectin epitopes have successfully been unmasked in transverse sections of wheat grain by pre-treatments with xylanase and lichenase (Chateigner-Boutin et al., 2014). However, as shown in our example, these assays on resin sections are often not trivial, notably because the acrylic resin limits the accessibility of both the enzyme and the antibody to the sections and likely impedes the unmasking of cell wall polymers by enzymatic degradation. Due to the absence of embedding agent, both the enzyme and the probe may access the cell walls present in the inner surface of the sections cut with a microtome or a vibratome and the thickness of the samples cut with a microtome seems ideal to observe strongly contrasted labeling between sections treated with or without cell wall degrading-enzymes. However, the distinct labeling obtained between the wax-embedded and the agarose-coated sections when they are pre-treated with pectate lyase might reflect some limitations to the preparation of plant samples with a microtome. Indeed, unlike the sections cut with a vibratome, no microdomains are detected when the wax-embedded sections are pre-treated with a pectate lyase and labeled with the LM19 antibody (Figure 5D). This difference might be due to the loss of tissue integrity that occurs during the steps of dehydration and infiltration. It is also possible that these microdomains still detectable with the LM19 antibody after the action of pectate lyase (Figure 5F) are a consequence of the thickness of the samples. However, several arguments go against this hypothesis. By contrast with wax-embedded sections that are attached to a slide, the vibratome-sections are free in solution. It makes the sections more readily subject to the enzymatic treatment. Likewise, if the enzyme concentration had been too low to completely degrade homogalacturonan, we would have expected to observe random patchy pattern of detection instead of a consistent detection that occurs in specific microdomains such as the corners of the cell corners. Overall, our understanding is (i) that the acrylic resin diminishes the accessibility of enzymes and probes to their substrates and their targets, respectively, (ii) that microtome-cut sections are useful to observe contrasted labeling when the sections are subjected to enzymatic pre-treatments, and (iii) that sectioning plant material with a vibratome increases the probability to detect microdomains that are protected from enzymatic degradation.

**Figure 7:**
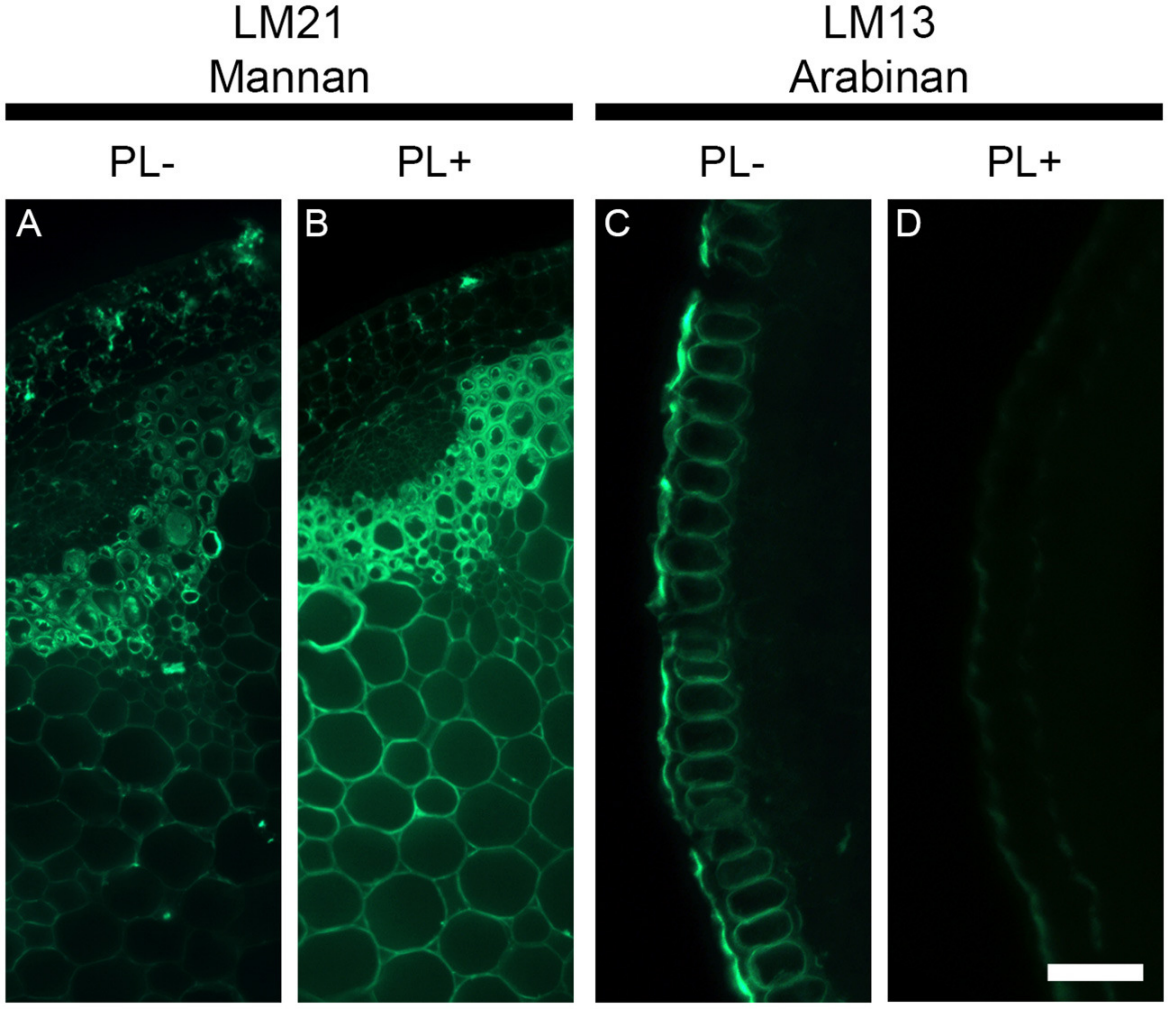
The deconstruction of plant cell walls by enzymatic degradation can have opposite effect on epitope detections. The figure shows the effect of a pectate lyase pretreatment on the detection of the LM21 mannan epitope and the LM13 pectic arabinan epitope in transverse stem section of *A. thaliana* cut with a vibratome. Whereas the pectate lyase pretreatment (PL+) results in an increased detection of the LM21 epitope, it leads to a reduced detection of the LM13 epitope. **(A,C)** Sections pretreated with phosphate buffer (pH 7.2) (PL−). **(B,D)** Sections pretreated with a recombinant pectate lyase in CAPS buffer (pH 10) (PL+). A and B are transverse stem sections immunolabeled with the LM21 antibody. **(C,D)** show the immunolabeling of transverse epidermal sections with the LM13 antibody. Scale bar: **(A,B)**: 100 μm, **(C,D)**: 55 μm.

Unmasking of cell wall epitopes by enzymatic pre-treatment might not always exclusively attribute to the polymer targeted by the enzyme. Cell wall polysaccharides are entangled together and the degradation of a specific polymer can sometimes be accompanied by the extraction of other polymers. To exemplify this, we used pectate lyase to degrade pectic homogalacturonan and two antibodies that recognized epitopes from distinct class of polysaccharides. The LM21 antibody binds to a mannan epitope that belongs to hemicelluloses and the LM13 antibody recognizes an epitope of arabinans from the class of pectins. Arabinans are part of rhamnogalacturonan I, which is covalently linked with homogalacturonan and both polysaccharides have frequently been shown to be tightly associated in plant cell walls (Jones et al., 2005). We have performed a pectate lyase pre-treatment on transverse stem sections of *A. thaliana* cut with a vibratome (Figure 7) and focused only on the epidermal cell walls for the LM13 immunolabeling as it has been shown that the LM13 antibody only binds consistently to this region (Verhertbruggen et al., 2013). By comparison with an untreated section (Figure 7A), the removal of pectic homogalacturonan with pectate lyase (Figure 7B) leads to an increased detection of the LM21 mannan epitope in the entire section, including the epidermis. By contrast, the detection of the LM13 epitope in the epidermal cell walls (Figure 7C) is severely reduced after a pectate lyase pre-treatment (Figure 7D). This suggests the action of pectate lyase indirectly leads to the release of the LM13 arabinan epitope. As shown by Marcus et al. (2008), the release of the outer homogalacturonan-rich layer by the action of pectate lyase provides a greater accessibility of the antibodies to epitopes that are hidden by this outer layer. The indirect removal of the polymers attached to this layer, such as rhamnogalacturonan I and their arabinans, might significantly contribute to the unmasking of epitopes. In the shown example, we used a highly purified recombinant enzyme that belongs to CAZY family PL10, a family well-known to specifically degrade homogalacturonan (Mayans et al., 1997). In many cases, cell wall degrading enzymes can have side activities, which obviously can lead to unexpected results. Consequently, the results obtained from enzymatic pre-treatments must be carefully interpreted.

## Conclusions

Here, we have compared three methods of sample preparation to study plant cell walls by light and fluorescence microscopy. The sample preparations using an ultramicrotome, a microtome or a vibratome offer distinct advantages and disadvantages and, as highlighted in Table 4, the convenience of these methods for microscopy depends of what is studied and the experimental aims. Ultramicrotome sectioning is particularly advised for hard and/or small specimens and when the analysis of the plant material by electron microscopy is planned. From our experience, the use of a microtome appears to be the best option for studies that include co-localization of epitopes as well as chemical and enzymatic pre-treatments. However, whenever possible, we advise beginning with the use a vibratome as the technique is fast, easy to master, offers the possibility to study large specimens, large number of biological replicates and is the best method to preserve the antigenicity of the plant material.

**Table 4:** Comparison of three methods of sample preparation applied to study plant cell walls by microscopy.

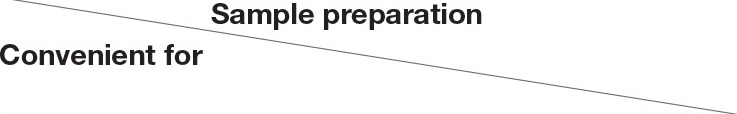	**Resin embedding with ultramicrotome sectioning**	**Wax embedding with microtome sectioning**	**No or agarose coating with vibratome sectioning**
1. Producing thin sections	+++	++	+
2. Obtaining a good proportion of sections with high quality	+++	+	++
3. Quickly producing sections	+	++	+++
4. Studying large specimens	+	++	+++
5. Studying large number of specimens	+	++	+++
6. Studying small or hard materials	+++	+	+
7. Studying primary fluorescence (based on its abundance) (see Figure 3)	+	++	+++
8. Immunolabeling of cell wall polysaccharides (based on the preservation of antigenicity) (see Figures 3–5)	+	++	+++
9. Performing co-localization study of epitopes (based on the ability to obtain thin sequential sections that have a well-preserved antigenicity)	++	+++	+
10. Applying enzymatic or pH pre-treatments (see Figures 5, 6)	+	+++	++
11. Examining samples by both light and electron microscopy	+++	−	−

*A triple + indicates the most convenient method to choose for an application, ++ indicates the second best method, + indicates the methods that is the less convenient for an application. A – means the technique is incompatible with the application*.

## Author contributions

Original idea: YV. Experimental work: YV (performed most of the experiments presented in the MS), JW (contributed to the preparation of plant material for immunomicroscopy and performed immunolabelings), HS supervised the work at LBNL. Writing of the manuscript: YV, FG, HS.

### Conflict of interest statement

The authors declare that the research was conducted in the absence of any commercial or financial relationships that could be construed as a potential conflict of interest.

## Abbreviations

BA-PBS: Blocking agent in phosphate-buffered saline solution
CAPS: 3-(cyclohexylamino)propanesulfonic acid
dH_2_O: distilled water
FITC: fluorescein isothiocyanate
LM: Leeds monoclonal
LR: London White resin
PBS: Phosphate-buffered saline solution
PEM buffer: 50 mM piperazine-*N,N'*-bis[2-ethane-sulfonic acid], 5 mM methylene glycol bis(β-aminoethylether)-*N,N,N',N'*-tetraacetic acid, 5 mM MgSO4 (pH 6.9)
PTFE: polytetrafluorethylene
ROI: Region of interest
RT: Room temperature.
