# Supplementary figures and images for: A Comparative Study of Sample Preparation for Staining and Immunodetection of Plant Cell Walls by Light Microscopy
Source: Front Plant Sci. 2017 Aug 29;8:1505. doi: 10.3389/fpls.2017.01505 (PMC5581911; doi:10.3389/fpls.2017.01505)

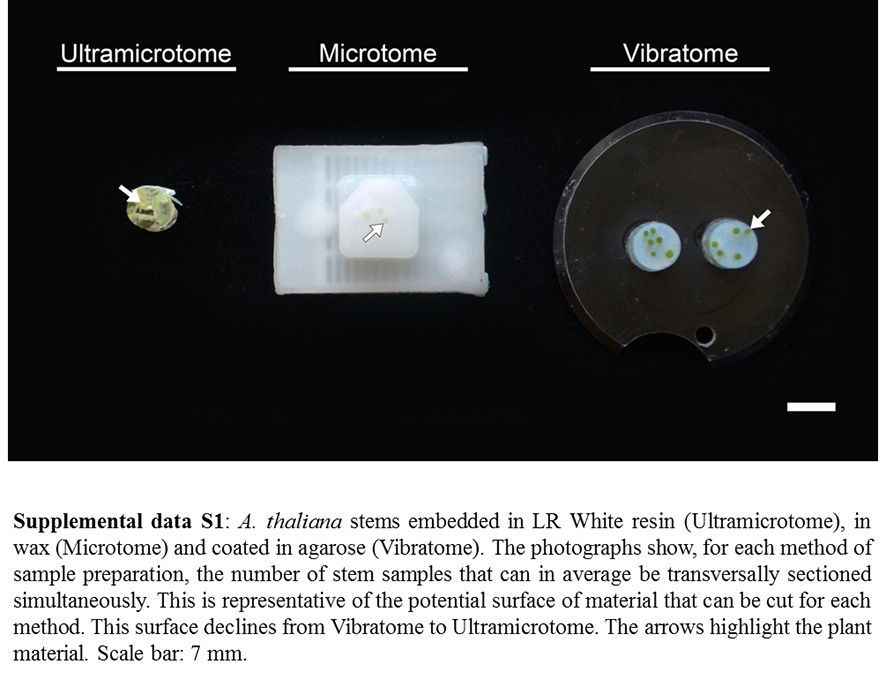

Supplement: Supplementary file 1 [file Image1.TIF]

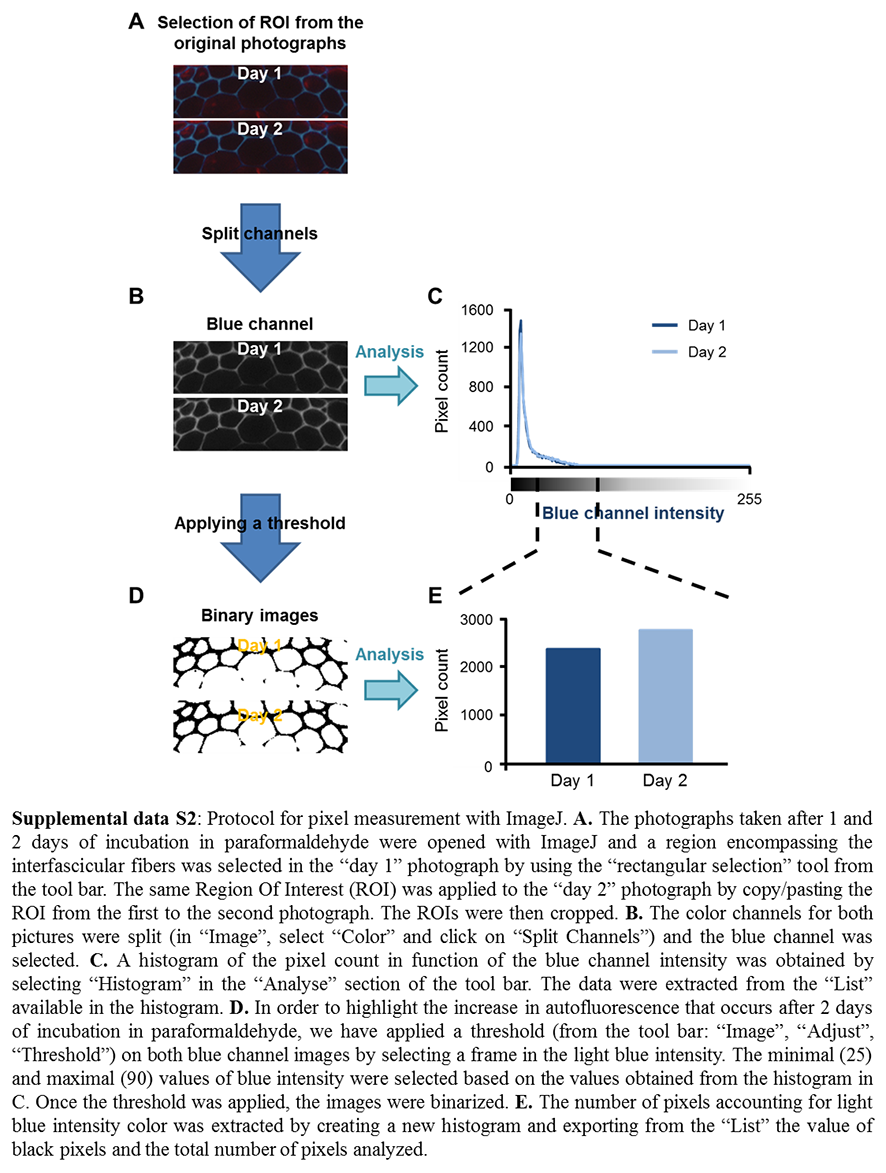

Supplement: Supplementary file 2 [file Image2.TIF]

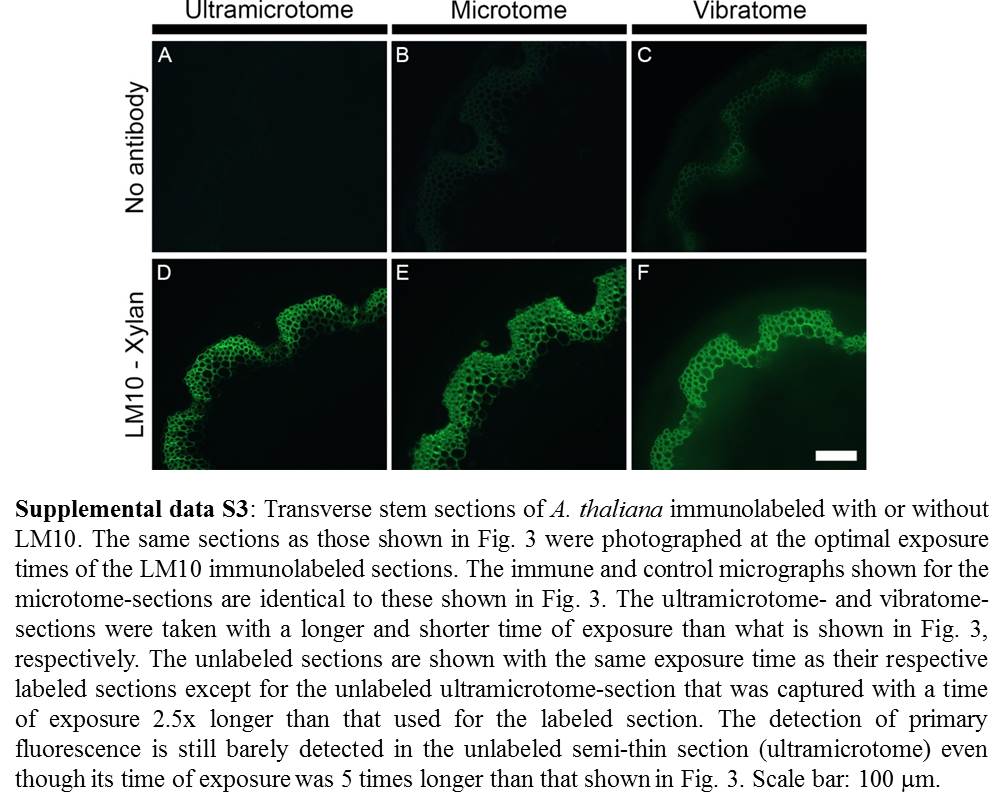

Supplement: Supplementary file 3 [file Image3.JPEG]

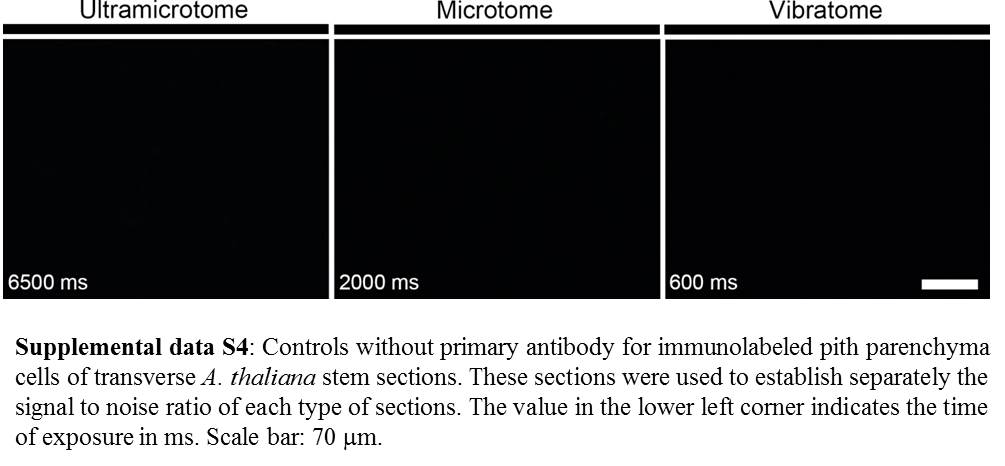

Supplement: Supplementary file 4 [file Image4.JPEG]
